# Carcinogenic Parasite Secretes Growth Factor That Accelerates Wound Healing and Potentially Promotes Neoplasia

**DOI:** 10.1371/journal.ppat.1005209

**Published:** 2015-10-20

**Authors:** Michael J. Smout, Javier Sotillo, Thewarach Laha, Atiroch Papatpremsiri, Gabriel Rinaldi, Rafael N. Pimenta, Lai Yue Chan, Michael S. Johnson, Lynne Turnbull, Cynthia B. Whitchurch, Paul R. Giacomin, Corey S. Moran, Jonathan Golledge, Norelle Daly, Banchob Sripa, Jason P. Mulvenna, Paul J. Brindley, Alex Loukas

**Affiliations:** 1 Centre for Biodiscovery and Molecular Development of Therapeutics, Australian Institute of Tropical Health and Medicine, James Cook University, Cairns, Queensland, Australia; 2 Department of Parasitology, Faculty of Medicine, Khon Kaen University, Khon Kaen, Thailand; 3 Department of Microbiology, Immunology and Tropical Medicine, and Research Center for Neglected Diseases of Poverty, George Washington University, Washington, D.C., United States of America; 4 The Institute for Molecular Bioscience, The University of Queensland, Brisbane, Queensland, Australia; 5 Microbial Imaging Facility, The iThree institute, University of Technology Sydney, Ultimo, New South Wales, Australia; 6 Queensland Research Centre for Peripheral Vascular Disease, Australian Institute of Tropical Health and Medicine, James Cook University, Cairns, Queensland, Australia; 7 Department of Vascular and Endovascular Surgery, The Townsville Hospital, Townsville, Queensland, Australia; 8 Department of Pathology, Khon Kaen University, Khon Kaen, Thailand; 9 QIMR Berghofer Medical Research Institute, Brisbane, Queensland, Australia; New York University, UNITED STATES

## Abstract

Infection with the human liver fluke *Opisthorchis viverrini* induces cancer of the bile ducts, cholangiocarcinoma (CCA). Injury from feeding activities of this parasite within the human biliary tree causes extensive lesions, wounds that undergo protracted cycles of healing, and re-injury over years of chronic infection. We show that *O*. *viverrini* secreted proteins accelerated wound resolution in human cholangiocytes, an outcome that was compromised following silencing of expression of the fluke-derived gene encoding the granulin-like growth factor, *Ov*-GRN-1. Recombinant *Ov*-GRN-1 induced angiogenesis and accelerated mouse wound healing. *Ov*-GRN-1 was internalized by human cholangiocytes and induced gene and protein expression changes associated with wound healing and cancer pathways. Given the notable but seemingly paradoxical properties of liver fluke granulin in promoting not only wound healing but also a carcinogenic microenvironment, *Ov*-GRN-1 likely holds marked potential as a therapeutic wound-healing agent and as a vaccine against an infection-induced cancer of major public health significance in the developing world.

## Author Summary

The oriental liver fluke *Opisthorchis viverrini* infects millions of people in SE-Asia and kills 26,000 people each year due to parasite-induced liver cancer. The mechanisms by which the parasite causes cancer are complex, but a role for excessive wound healing in response to feeding parasites in the bile ducts has been proposed. We show that a growth factor (granulin) secreted by the worm gets into bile duct cells and drives wound healing and blood vessel growth. We delve into this “supercharged” wound healing process and uncover a range of signaling molecules that initiate healing, but when dysregulated, can result in a deadly liver cancer. On the upside, this liver fluke growth factor is now a candidate drug for the development of novel wound healing therapeutics to treat chronic wounds, such as diabetic ulcers. Understanding this process is another step on the road to developing a vaccine to reduce both parasite burdens and the incidence of the most prevalent and fatal cancer in Thailand and surrounding countries.

## Introduction

Approximately 10 million people in Thailand and Laos are infected with the South East Asian liver fluke *Opisthorchis viverrini* [1,2]. Infection with *O*. *viverrini*, a one-centimeter long flatworm that inhabits the bile ducts, is strongly associated with the induction of cholangiocarcinoma (CCA), cancer of the bile ducts [3]. The World Health Organization’s International Agency for Research on Cancer classifies infection with *O*. *viverrini* as a ‘group 1 carcinogen [1,3,4,5]. In Thailand and neighboring countries, cyprinid fish that are intermediate hosts for *O*. *viverrini* are eaten raw as a staple of the diet [1,2]. Infected individuals in endemic areas suffer the world’s highest incidence of CCA, 65 times that experienced in non-endemic regions, and accounting for up to 81% of liver cancers in this region [3,4]. CCA is a primary cancer originating in cholangiocytes, the epithelial cells that line the biliary tree. It has long latency, is invasive, metastasizes, is relatively non-responsive to anti-tumor agents and has a dismal prognosis.

How opisthorchiasis induces cholangiocarcinogenesis is likely multi-factorial, involving immunopathogenesis, increased consumption of dietary carcinogens, and the secretion of parasite proteins mitogenic for cholangiocytes [2]. We described a liver fluke-derived homologue of the human growth factor granulin, termed *Ov*-GRN-1, from the excretory/secretory (ES) products of *O*. *viverrini* [2,6,7]. *Ov*-GRN-1 binds to cholangiocytes in experimentally infected hamsters and stimulates proliferation of fibroblasts and CCA cell lines. Here we sought to determine whether *Ov*-GRN-1 possesses wound healing capacity and might therefore function to repair the chronic damage it causes in the bile ducts during feeding activity and the ensuing chronic inflammation. Moreover, given the physiologic and genetic similarities between chronically healing wounds and cancer [8], we sought to address whether *Ov*-GRN-1 promotes cellular changes that are conducive to the establishment of a tumorigenic environment.

## Results

### *Ov*-GRN-1 is internalized by cholangiocytes

**Fig 1:**
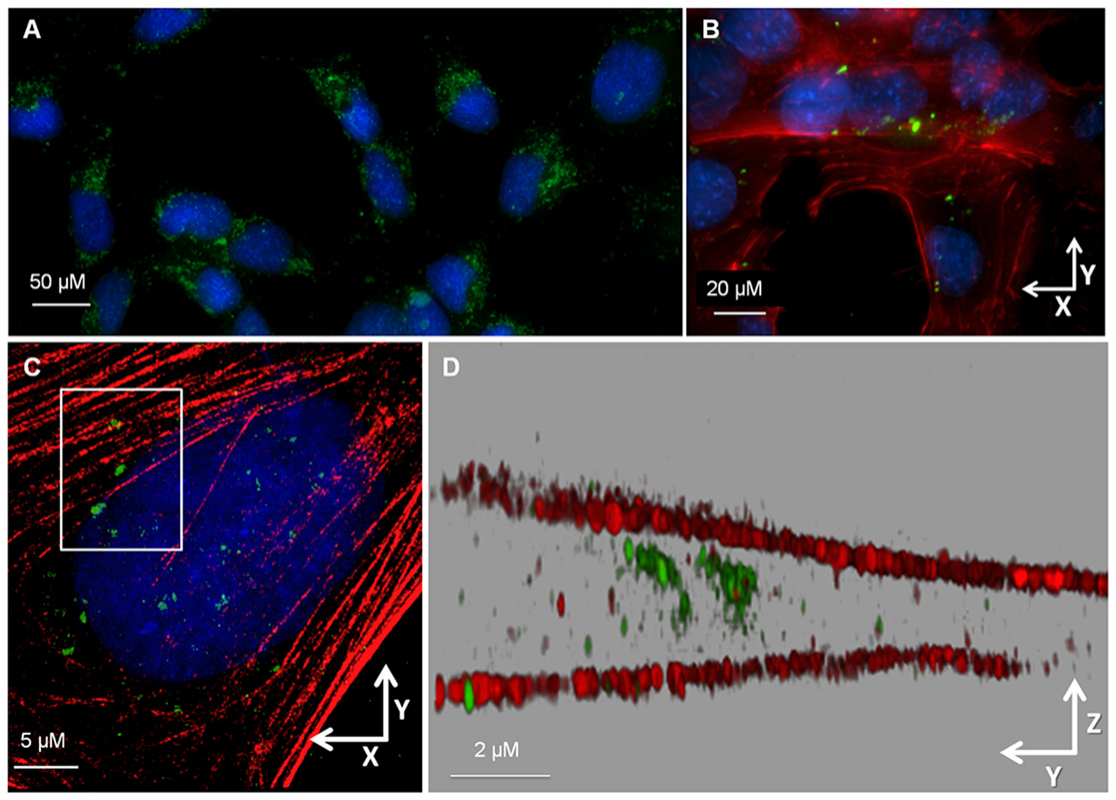
Liver fluke granulin internalized by H69 cholangiocytes. **(A)** Widefield (deconvolved) micrographs showing the lateral (xy) overview of live H69 cholangiocytes imaged after 18 h incubation with Alexa Fluor 488-conjugated r*Ov*-GRN-1 (green) and Hoescht nuclear stain (blue). **(B)** With further magnification of fixed cells the labeled r*Ov*-GRN-1 was evident among the cytoskeletal actin network (red) of numerous cells with DAPI (blue) stained nuclei. **(C)** 3D-SIM lateral (xy) overview image of a well-separated individual cholangiocyte stained as in panel B. **(D)** Rendered axial (yz) view of boxed inset in (C) showing r*Ov*-GRN-1 (green) present between the apical and basal actin filaments (red) of the cholangiocyte (DAPI channel omitted). Additional material shown in S1 Fig and S1 Movie.

Using fluorescence microscopy we report that recombinant *Ov*-GRN-1 (r*Ov-*GRN-1) labeled with Alexa Fluor 488 (AF) was putatively internalized by ~75% of cells from an immortalized human cholangiocyte cell line, H69 (Fig 1A and 1B, S1A–S1D Fig). Cholangiocytes co-cultured with r*Ov*-GRN-1-AF exhibited significantly higher (*P* < 0.001) per cell fluorescence intensity (6.4-fold, or 15.3-fold RFU/mole) than cholangiocytes co-cultured with a control recombinant protein (thioredoxin-AF, rTRX-AF) that had been expressed and purified under identical conditions (S1E Fig). Using 3D-structured illumination microscopy, r*Ov*-GRN-1-AF was detected between the apical and basal actin filaments of cells in monolayer, confirming internalization in cholangiocytes of the liver fluke granulin (Fig 1C and 1D, S1 Movie). The precursor of human granulin is expressed as a seven-domain granulin unit, known as progranulin (PGRN), and initiates context-dependent autocrine and paracrine signaling cascades [9,10,11,12]. PGRN is internalized by cells and targeted to a specific organelle, commonly lysosomes, when bound to co-factors such as sortilin or CpG nucleic acid motifs [9,10,11,13]. Attempts to identify the sub-cellular location of r*Ov*-GRN-1 after internalization by cholangiocytes using a range of organelle-specific markers suggested a cytosolic location, as specific co-localization to organelles was not apparent (S2 Fig). The lack of involvement of an organelle suggested direct cell entry followed by interactions with signaling cascades, rather than the more conventional growth factor receptor-based signal initiation. While unusual, direct cell entry and interaction with signaling molecules is known for small growth factors with alkaline tails, such as basic FGF [14,15]; the C-terminus of *Ov*-GRN-1 is highly basic [7] with a predicted pI of 12, characteristics that also support this mode of cell entry.

### Silencing of *Ov-grn-1* expression impairs parasite-driven wound healing

**Fig 2:**
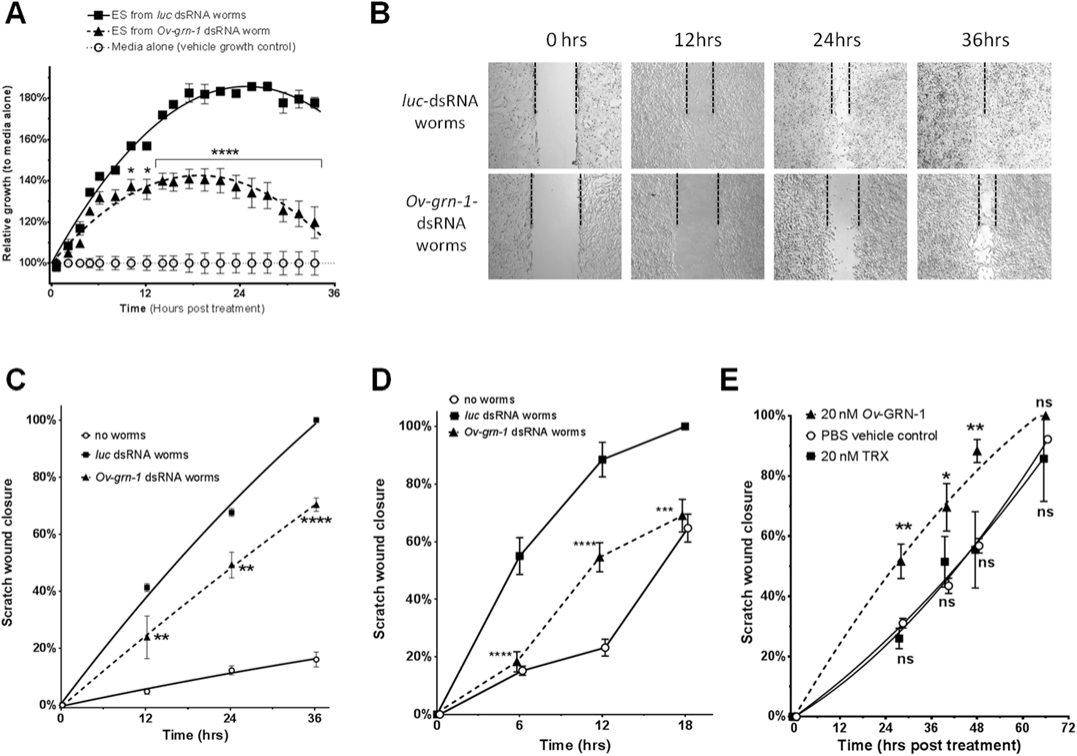
*Ov-*GRN-1 stimulated wound repair *in vitro*. **(A)** Cholangiocytes exposed to ES products from flukes where *Ov-grn-1* had been silenced by RNA interference displayed significantly reduced proliferation over 36 h of co-culture. ES products (10 μg/ml) were derived from flukes that were exposed to dsRNAs for 5 days. Cell proliferation was monitored in real time using xCELLigence; every tenth data point is shown to aid visualization. Statistical comparisons were between *Ov-grn-1-* and *luc-*dsRNA-treated parasites. **(B)** Images of the scratch assay involving H69 cholangiocyte monolayers co-cultured in Transwell plates with *Ov-grn-1* or *luc*-dsRNA-treated. Dotted lines denote wound edges over time. **(C)** Selected time points were measured from the photographs in (B); statistical comparisons were between cells cultured with *Ov-grn-1-*and *luc*-dsRNAs. **(D)** Wound healing scratch assay as shown in panel c but using the CCA cell line M214 (D). **(E)** Wound healing scratch assay as shown in panels (C) and (D) but recombinant protein applied to cells instead of co-culturing cells with live flukes. Statistical comparisons were between 20 nM rTRX and r*Ov*-GRN-1 treatments or rTRX and PBS treatments. For all panels, data points represent the averages of two or three biological replicates with 3–5 biological replicates displayed with SEM error bars (some bars masked by data points). *P<0.05, **P<0.01, ***P<0.001, ****P<0.0001, ns = not significant. Additional data shown in S2 Fig.

Previously, we silenced expression of the *Ov-grn-1* gene using RNA interference (RNAi) that reduced cell proliferation of cholangiocytes co-cultured with the liver flukes [16]. To address the role of *Ov*-GRN-1 in wound repair we silenced expression of *Ov-grn-1* using RNAi and assessed the ability of ES products from dsRNA-treated flukes to accelerate cell proliferation and wound repair. Levels of mRNA encoding *Ov*-GRN-1 were depleted by 97% in worms transduced with dsRNA specific for *Ov-grn-1* but not affected by control dsRNA specific for *luciferease* (*luc*) (S3 Fig). ES products were collected from culture supernatants of dsRNA-treated flukes and effects of the ES on proliferation of cholangiocytes assessed. ES products collected on days 1, 5 and 7 from *Ov-grn-1*dsRNA-treated flukes reduced cell proliferation by ~48% (*P* < 0.01; F_(DFn, DFd)_ = 24.27 _(3,7)_) compared to ES from *luc*-treated flukes (Fig 2A and S3 Fig). To ensure that *Ov-grn-1*-dsRNA treatment did not have a major impact on the ES composition of the flukes, we compared ES profiles from *Ov-grn-1*- and *luc*-dsRNA treated flukes by SDS-PAGE, and did not detect obvious differences in protein yield or composition (S4 Fig).

At the outset, we assessed the role of *Ov-*GRN-1 in wound repair using *in vitro* scratch assays given that the procedure is a facile surrogate of cell migration and wound closure [17]. dsRNA-treated flukes were co-cultured in Transwell plates such that they were separated from the underlying cells by a porous membrane, but ES products could traverse the inner membrane of the chamber. Firstly, we showed that ES products from *luc* dsRNA-treated flukes substantially accelerated wound healing compared to both cholangiocytes and CCA cell lines that were not co-cultured with flukes (Fig 2C and 2D). Secondly, and pivotal to this study, significantly less wound healing/closure was induced by *Ov-grn-1* dsRNA-treated flukes in both cholangiocytes over 36 hours (*P* < 0.01–0.0001; Fig 2B and 2C) and CCA cells over 18 hours (*P* < 0.001–0.0001; Fig 2C) than with control *luc* dsRNA-treated flukes. Fewer cells crossed the margin of the wound of the scratched monolayers cultured with ES products from *Ov-grn-1* dsRNA-treated flukes at the early time points (6–12 h, Fig 2B–2D), suggesting the involvement of cell migration in scratch closure rather than closure due simply to cell proliferation [17].

To confirm the role of *Ov*-GRN-1 in *in vitro* wound healing 20 nM r*Ov*-GRN-1 was shown to be sufficient to significantly accelerate healing of a cholangiocyte monolayer compared to cells exposed to control protein (rTRX) (F_(DFn, DFd)_ = 16.32_(2,33)_; *P* < 0.01) (Fig 2E).

### r*Ov*-GRN-1 accelerates wound healing in mice

**Fig 3:**
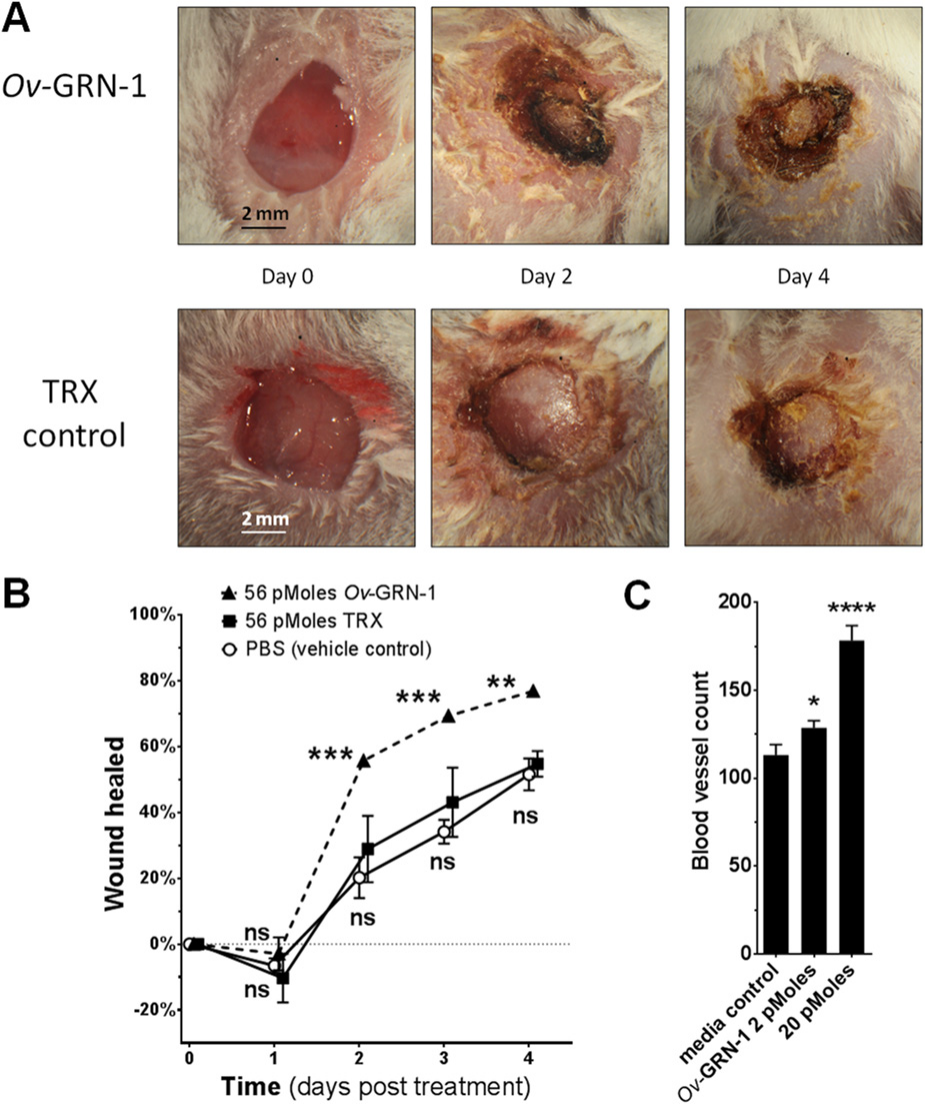
*Ov-*GRN-1 stimulated wound repair *in vivo*. **(A)** Sequential images over four days of healing wounds revealed the response of mice to treatment with recombinant r*Ov-*GRN-1 or rTRX control; skin-deep wounds made with a 5 mm diameter biopsy punch between the ears of Balb/c mice. Minor modifications (brightness, contrast, cropping) were made to aid viewing. **(B)** The rate of wound healing over four days was measured; wound closure was determined electronically from photographs by measuring wound areas with ImageJ software. To aid viewing, curves have been shifted left or right marginally to minimize error bar overlap. **(C)** Assessment of the angiogenic properties of recombinant *Ov*-GRN-1 in the chorioallantoic membrane (CAM) assay. The numbers of blood vessels in quail eggs that grew on 0.5 cm^2^ filter paper soaked in r*Ov*-GRN-1 or vehicle (control) were ascertained after incubation for 15 hours. Data points are the averages of two experiments with 3–5 biological replicates displayed with SEM bars. * = *P*<0.05, ** = *P*<0.01, *** = *P*<0.001, **** = *P*<0.0001, ns = not significant.

To determine whether r*Ov*-GRN-1 could accelerate wound repair *in vivo*, sub-cutaneous deep lesions were surgically inflicted between the ears on laboratory mice, treatment applied and the injury covered with spray plaster, after which the rate of wound healing was quantified at intervals of 24 hours for four days [18] (Fig 3A). This method is considered to be superior to the conventional abdomen wound protocol when investigating growth factors, since it quantifies healing primarily from epithelial re-growth rather than skin contraction [18,19]. Daily application of 56 pMoles of r*Ov-*GRN-1 significantly accelerated wound healing within 2–4 days compared wound closure in response to application of a control protein (rTRX) (F_(DFn, DFd)_ = 32.08 _(2,16)_; *P* < 0.01–0.001) or PBS (Fig 3B).

### Liver fluke granulin is angiogenic

Angiogenesis is an integral aspect of wound healing, is essential for the vascularization of new tissue, and is a cardinal hallmark of carcinogenesis. The chorioallantoic membrane (CAM) assay is a commonly accepted *in vivo* model of vertebrate angiogenesis [7,20,21]; moreover, the ancestral lineage of the granulin family of growth factors [22] made us conclude that the CAM assay was a suitable mean by which to assess angiogenic properties of *Ov*-GRN-1. Quail eggs were implanted with r*Ov*-GRN-1- or PBS-soaked membranes. Membranes with two picomoles (*P* < 0.05) or 20 picomoles (*P* < 0.0001) of r*Ov*-GRN-1 induced angiogenesis (F_(DFn, DFd)_ = 108.4_(2,9)_) (Fig 3C) in the embryo developing within the egg.

### r*Ov*-GRN-1 induces changes in cholangiocyte protein and gene expression associated with wound healing and cancer

**Fig 4:**
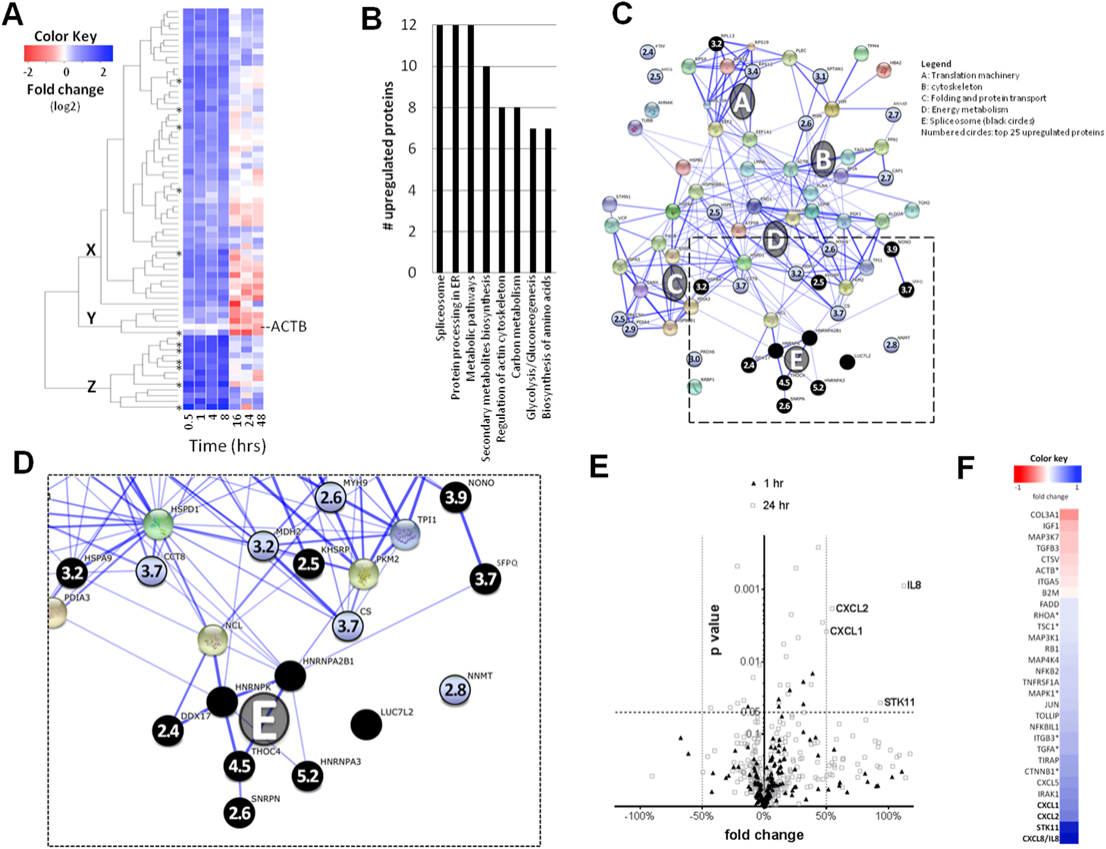
*Ov-*GRN-1 stimulated responses in cholangiocytes. **(A)** Clustered heatmap of 70 proteins for which expression levels were modulated between 0.5–48 h post-r*Ov*-GRN-1 treatments. Only proteins that underwent significant changes in expression levels (> ±1.5-fold change relative to time point zero) are shown. The Euclidean distance clustering grouped proteins by translational temporal patterns and the XYZ designations distinguish the three major regulation patterns. Asterisks denote spliceosome-associated proteins. **(B)** Major KEGG pathways of cholangiocyte proteins whose expression levels were modulated by r*Ov*-GRN-1. **(C)** Interactome of proteins whose expression levels were modulated by r*Ov-*GRN-1. The top 25 proteins in terms of expression level changes were numbered with the average fold-upregulation; spliceosome-associated proteins shown surrounded by black circles. The A-E designations signify the major gene ontology groupings (defined in the legend) and the thickness of the lines linking different proteins represents the strength of the interactions. **(D)** Magnification of the boxed spliceosome grouping “E” from panel C. **(E)** Volcano plot of the cholangiocyte gene response to co-culture with r*Ov*-GRN-1. Gene expression was measured using gene arrays designed to assess wound healing, oncogenesis, EMT and TLR associated transcripts detected using qPCR. **(F)** Heatmap depicting the changes of the significantly regulated (*P* < 0.05) genes shown in (E). Genes modulated with a > ±1.5-fold change denoted using bold type. Asterisks denote genes whose expression level significantly changed within one hour of co-culture with r*Ov*-GRN-1. Genes where expression levels underwent significant changes within 24 hours (but not by one hour) of co-culture are indicated by the absence of asterisks. The color keys for panels (A) and (F) represent fold-change proportional to color intensity. Complete data sets of proteome and transcriptome changes provided in S1–S4 Tables.

We employed isobaric tags for relative and absolute quantitation (iTRAQ) of changes in expression of cholangiocyte proteins induced by r*Ov*-GRN-1. Using the Scaffold program, we reliably validated 215 proteins in cholangiocytes identified by Mascot compared to cells at baseline and at subsequent intervals (S1 Table). r*Ov-*GRN-1 induced >50% change in detectable expression levels (*P* < 0.05) of 70 cholangiocyte proteins at ≥1 time point compared to control cells (Fig 4A and S2 Table). During co-culture of up to eight hours there was substantial up-regulation of protein expression, after which moderation or down regulation of the proteins ensued beyond 16 hours from the start of the analysis (Fig 4A). Three KEGG pathways with 12 significantly regulated proteins each—the spliceosome, endoplasmic reticulum protein processing and metabolic pathways (Fig 4B) were revealed by protein ontology analysis in the cholangiocytes cultured with the parasite granulin. Euclidean distance clustering revealed the internal patterning of temporal translational changes (Fig 4A), where group X proteins underwent a short-term up-regulation (0.5–8 h) followed by a lessening of expression. Group Y proteins also underwent a short-term up-regulation followed by a substantial down-regulation. Group Z proteins were distinct due to their high and rapid short-term up-regulation. Notably, six of the 13 group Z proteins are associated with the spliceosome (Fig 4A and 4B). The dysregulated proteins were subjected to a network analysis (Fig 4C). When the top-25 most highly up-regulated proteins were considered, proteins involved in the spliceosome pathway were most highly represented (Fig 4D), and included the top three (HNRNPA3, THOC4 and NONO) and nine of the top 25 most highly up-regulated proteins.

Mass spectrometry is constrained in its ability to characterize changes in low abundance proteins such as growth factors and cytokines. We therefore assessed the changes in cholangiocyte gene expression after one and 24 hours of co-culture with r*Ov-*GRN-1 using gene arrays targeting epithelial to mesenchymal transition (EMT), oncogenesis, wound healing and Toll-like receptor signaling (S3 Table). Thirty genes underwent an *Ov-*GRN-1-induced change (*P* < 0.05) in regulation (Fig 4E and S4 Table), including four which exhibited >50% change in expression levels. Three of the four upregulated genes encoded proteins from the C-X-C ligand chemokine family of cytokines: *cxcl1*, *cxcl2* and *cxcl8* (also known as*interleukin-8*); the fourth gene encoded for *serine/threonine kinase 11* (*stk11)*, also known as *liver kinase B1*. Another member of the *cxcl* family, *cxcl5*, was significantly upregulated, but fell below the 50% cutoff (43%).

## Discussion

We report for the first time the secretion of a growth factor from a metazoan pathogen that promotes wound healing of mammalian host tissue *in vivo*. The implications of the findings are multi-fold and significant. Firstly, the instrumental role described here for *Ov*-GRN-1 in orchestrating wound repair implies that this protein represents an attractive target for the development of a vaccine that thwarts regulation of the microenvironment within the biliary tract parasitized by the liver fluke. Indeed we previously showed that antibodies to r*Ov*-GRN-1 block proliferation of cholangiocytes [7], which further bolsters the proposition of a vaccine with both anti-infection and anti-cancer properties. One potential caveat of a vaccine that blocks wound repair however is the consequences of an aggressive inflammatory response in the absence of wound resolution, including uncontrolled sepsis or other complications, so appropriate consideration is warranted. Second, the findings highlight the potential therapeutic application of *Ov*-GRN-1 as a novel biologic for treating both acute and chronic wounds, such as recalcitrant ulcers on the extremities of diabetic patients [23].

Mammalian granulins play diverse roles continuously during development from the embryo into adult life, including key roles in tissue remodeling and inflammation [22]. Mutations in the human granulin gene result in a spectrum of conditions, including neurodegenerative disorders [24] and malignant growth and metastasis [25]. Indeed, granulin has a central role in carcinogenesis of a range of malignancies [22]; pertinent to our findings, granulin is over-expressed in hepatocellular carcinoma (HCC) [26] and renders HCC cells resistant to Natural Killer cell-mediated cytotoxicity by modulating expression of MHC-associated genes [27]. By contrast, granulins of pathogens have received little attention. We detected *O*. *viverrini* granulin (*Ov*-GRN-1) in the ES products of adult flukes and provided the first evidence of a parasite growth factor that drove proliferation of host cells [6,7]. The recent report of the *O*. *viverrini* genome revealed additional members of the granulin family–a single granulin domain protein (*Ov*-GRN-2) and a pro-granulin (PGRN) containing eight granulin subunits [28]. Products of either of these genes were not evident within the ES proteome [6] and their role in the host-parasite relationship is unclear.

The mechanisms by which vertebrate or liver fluke granulins drive cell proliferation and wound repair are poorly understood. Vertebrate PGRN contains seven individual granulin subunits that are post-translationally processed. Mouse PGRN but not the individual subunits of granulin binds to TNF receptors (TNFR), and antagonizes TNF signaling [29]. Dissimilar to PGRN, *Ov*-GRN-1 consists of a signal peptide and a single granulin motif [7]. Although the ability of r*Ov*-GRN-1 to bind to TNFR has not been investigated, probing a microarray of the human proteome microarray [30] with labeled r*Ov*-GRN-1 failed to reveal binding to any isoforms of TNFR, or indeed to any other obvious cell surface receptors, on the array.

Cholangiocyte proteins involved in the spliceosome pathway were significantly regulated after exposure to r*Ov*-GRN-1 *in vitro*. The majority of intron removal from pre-RNA molecules is catalysed by the spliceosome, a large ribonucleo-protein complex that consists of five small nuclear ribonucleo-protein particles (snRNP, U1-6) and >150 other proteins [31]. One critical component of the wound healing process that is heavily regulated by RNA binding and splicing is the epithelial to mesenchymal transition (EMT), which increases the migratory and invasive properties of cells and thereby promotes wound closure [32,33,34]. However, cancerization also is an untoward consequence of EMT, and aggressive tumours often display dysregulated expression of spliceosome proteins [31,35].

Liver fluke granulin stimulated expression of genes encoding the chemokines CXCL1, 2, 5 and 8 (also known as IL-8). These chemokines signal through the receptor CXCR2 [36,37] by internal transactivation of the epidermal growth receptor (EGFR) and EGFR signaling through the mitogen activated protein kinase (MAPK) pathways [38]. Chemokines play central roles in wound repair, angiogenesis and recruitment of immune cells [36,39,40]. Inhibitors of MAPK signaling block r*Ov-*GRN-1-induced cell proliferation [7], and the increased expression of *cxcl* genes induced in cholangiocytes by r*Ov*-GRN-1 may underlie this observation. In addition, expression levels of transcripts encoding several kinases including *stk11* and *irak1* were markedly stimulated by the parasite granulin. Both STK11 (liver kinase B1) and IRAK1 (Interleukin-1 receptor associated kinase 1) control signaling in inflammatory pathways and regulate chemotaxis in diverse processes including wound healing [41,42]. Moreover, somatic mutations in *stk11* [43] and *irak1* [44] associated with malignancy. Upregulation of these kinases during proliferation of cholangiocytes within the liver fluke-parasitized biliary tree may, therefore, increase the likelihood of these mutations.

Topical application of picomoles of r*Ov*-GRN-1 significantly accelerated repair of wounds in the skin of mice. Although liver fluke granulin triggers changes in the cellular proteome that establish a pre-tumorigenic environment, short-term therapy would reduce the likelihood of inducing cancer in patients. Whereas advances in understanding the impaired angiogenesis in non-healing wounds have been reported, few effective agents that promote or expedite wound healing and closure are yet available [45]. The ability of r*Ov*-GRN-1 to accelerate wound healing in mice and promote angiogenesis *in vivo* revealed that this growth factor holds noteworthy promise for a new category of medicines for non-healing wounds and related indications.

Other growth factors are of interest for their therapeutic properties, notably human PGRN due to its ability to bind to TNFR. Indeed, recombinant human PGRN inhibits TNF-activated signaling and protected against inflammation in rodent models of arthritis [29]. PGRN further exerts its anti-inflammatory influence by inducing naïve T cells to transform into FOXP3-expressing regulatory T cells (Tregs) [46], a lymphocyte type that is underrepresented in inflammatory diseases but the presence of which is a hallmark of helminth infections [47,48]. Indeed we speculate now that *Ov*-GRN-1 may be the major inducer of Tregs during opisthorchiasis, but this hypothesis clearly warrants testing.

In conclusion, we have shown using gene silencing and recombinant protein technologies that the most carcinogenic of parasitic helminths, the liver fluke *O*. *viverrini*, secretes a growth factor which in isolation is sufficient to repair wounds both in monolayers of cultured human cholangiocytes and in the skin of mice. While our mouse cutaneous wound healing studies are informative and shed light on the potential therapeutic application of *Ov*-GRN-1 for chronic wounds, they do not directly address the role of the protein in host-fluke interactions in the biliary tree. With recent advances in genome editing using CRISPR-Cas9, we will soon be well placed to knock out the *Ov-grn-1* gene, facilitating *in vivo* studies that will specifically address the role of the protein in healing parasite-induced wounds in the bile ducts. *Ov*-GRN-1 therefore is a worthy candidate at which to target novel interventions—drugs and/or vaccines with both anti-helminth and anti-cancer activity. Moreover, *Ov*-GRN-1 offers potential as a novel biologic for treating acute and chronic wounds where normal tissue repair mechanisms are insufficient. Now more than ever, there is acute need for new therapeutics to combat the epidemic of inflammatory diseases, particularly diabetes and associated chronic ulceration. The therapeutic efficacy of parasitic helminths and their secreted products in treating inflammatory diseases is clear-cut [49]. The present findings indicate that parasite growth factors, which by their very nature have evolved to repair damaged tissues within their hosts, offer great promise as a novel therapeutic modality informed by millennia of host-parasite coevolution.

## Materials and Methods

### Ethics statements

#### Hamsters

Hamsters for *O*. *viverrini* lifecycle continuation were purchased from the animal facility, Faculty of Medicine, Khon Kaen University. Study design protocols and standard operating procedures adhered to and were approved by the Animal Ethics Committee of Khon Kaen University according to the Ethics of Animal Experimentation of the National Research Council of Thailand, approval number AEKKU43/2555.

#### Fish

Freshwater fish required for maintenance of the *O*. *viverrini* life cycle were purchased from a local market in the Muang District, Khon Kaen province, Thailand.

#### Mice

Mouse wounding studies were conducted in accordance with and approved by the James Cook University Small Animal Ethics Committee, approval number A1806.

#### Quail eggs

Eggs (age of embryonation < 7 days) were obtained from Quail Kingdom farm, Jimboomba, Queensland. Ethics applications were not required as the eggs were only used at the early stage with the minimum time required to complete laboratory procedures.

### Parasite culture

Excretory/secretory (ES) and somatic proteins were harvested from adult *O*. *viverrini* grown in laboratory hamsters as described [7,50]. Briefly, *O*. *viverrini* metacercariae harvested from naturally infected cyprinoid fish were used to infect hamsters (*Mesocricetus auratus*) by stomach intubation. Hamsters were euthanized three months after infection, when adult *O*. *viverrini* flukes were removed from the biliary tract. The flukes were washed and cultured in modified RPMI-1640 (Life Technologies) containing penicillin and streptomycin at 37°C/5% CO_2_ for three days. Culture supernatant was retained as ES products of the parasites, and stored at -80°C [50].

### Auto-induction of recombinant protein expression in *E*. *coli*

*Ov-grn*-1 pET41a or thioredoxin (*trx*) cDNAs contained within the pET32a (Novagen) plasmid were transfected into BL21 *Escherichia coli* cells (Life Technologies) and used to create recombinant protein with auto-induction as previously described [7,51]. Briefly, ZYM-5052 culture media was supplemented with 100 μM Fe(III)Cl_3_ and 100 μg L^-1^ kanamycin to produce recombinant protein (r*Ov-*GRN-1) or 50 μg L^-1^ ampicillin to produce rTRX [51]. Two hundred ml of inoculated media in a 1L baffled Erlenmeyer flask was incubated overnight at 37°C with 300 rpm rotation to induce expression with auto-induction.

### Recombinant granulin

Purification of r*Ov-*GRN-1 was achieved using an AKTA10 purification system at 4°C (GE Healthcare) [52]. The BL21 *E*. *coli* pellet was lysed with 3 freeze/thaw cycles followed by sonication (Q4000 sonicator, Qsonix) on ice. Twenty g of the resulting pellet was solubilized in 400 ml urea-containing nickel binding buffer (8 M urea/300 mM NaCl/50 mM imidazole/50 mM sodium phosphate pH 8 [Sigma]) at 4°C for 24 h with slow agitation. After filtration through 0.22 μM membranes, supernatants were incubated in nickel chelate resin on 2× 5 ml Histrap IMAC columns (GE Healthcare). The columns were washed in increasing concentration of imidazole (two column volumes [CV] at 50 mM/5 CV at 100 mM) after which bound material was eluted in 500 mM imidazole in binding buffer. The control rTRX protein was expressed and affinity purified similarly, but under native conditions (without chaotropes), as described [52].

### Protein refolding and purification

Refolding of urea-denatured r*Ov-*GRN-1 was performed with 28 mL of G10 Sephadex (GE) resin on a XK16/20 column (GE) [52]. A 120 ml Superdex 30 XK16/60 column (GE) was used to fractionate three ml of refolded r*Ov-*GRN-1 into 150 mM NaCl, 50 mM sodium phosphate, pH 6, at a flow rate of 1 ml min^-1^. Fractions containing r*Ov-*GRN-1 monomer eluting at a size equivalent of ~1 kDa were pooled. Protein concentration was established using a combination of the Bradford assay (Bio-Rad) and absorbance at 280 nm.

### Mammalian cell culture

The cholangiocyte cell line H69 is a SV40-transformed bile duct epithelial cell line derived from a non-cancerous human liver [53] and was obtained in 2010 from Dr. Gregory J. Gores, Mayo Clinic, Rochester, Minnesota. H69 cells and cells of the human cholangiocarcinoma (CCA) cell line KKU-M214 were maintained in T75 cm^2^ vented flasks (Corning) as monolayers as described [52,53,54,55] with minor modifications. KKU-M214 cells were maintained with regular splits using 0.25% trypsin (Life Technologies) every 2–5 days in complete media (RPMI with 10% fetal calf serum [FCS] and 1× antibiotic/antimycotic) at 37°C under 5% CO_2_. Cell proliferation assays were performed with low nutrient media containing 0.5% FCS. H69 cells were maintained under similar conditions with growth factor supplemented media [54] (DMEM/F12 with high glucose, 10% FCS, 1×antibiotic/antimycotic, 25 μg ml^-1^ adenine, 5 μg ml^-1^ insulin, 1 μg ml^-1^ epinephrine, 8.3 μg ml^-1^ holo-transferrin, 0.62 μg ml^-1^ hydrocortisone, 13.6 ng ml^-1^ T3 and 10 ng ml^-1^ EGF–Life Technologies). Low nutrient media for H69 cells was 5% complete media, i.e. 0.5% FCS and 5% of the growth factor concentrations for complete media. The identities as human-derived of both cell lines were confirmed with single tandem repeat (STR) analysis (15/15 positive loci across two alleles) and mycoplasma free at the DNA diagnostics centre (U.S.A.), accredited/certified by CAP, ISO/IEC 17025:2005 through ACLASS.

### Cell proliferation monitoring in real time

Cells were seeded at 1500 cells per well in 200 μl of complete media as described above in E-plates (ACEA Biosciences) and grown overnight while monitored with an xCELLigence SP system (ACEA Biosciences) which monitors cellular events in real time by measuring electrical impedance across interdigitated gold micro-electrodes integrated on the bottom of tissue culture plates [56]. Cells were washed three times with PBS and replaced with 180 μl of low nutrient media as described above and incubated for a minimum of 6 h before further treatments. Treatments were prepared at 10× concentrations and added to each well in a total volume of 20 μl. The xCELLigence system recorded cell index readings hourly for 5–6 days after treatment. Cell index readings were normalized before treatment and cell proliferation ratios were determined from biological triplicates and represent the relative numbers of cells compared to control cells.

### Cell scratch assay

H69 cells in complete media (see above) that were grown to confluence in 6 well plates (Falcon) were wounded by scratching the cell monolayer with a disposable 200 μl pipette tip, as described [17]. The wound in the monolayer was photographed regularly and closure was assessed using ImageJ software (National Institute of Health, U.S.A.). Wound widths over time were plotted and compared to controls with matched 2-way ANOVA and Dunnett’s correction for multiple comparisons. For cell scratch assays performed in co-culture with liver flukes, wounded monolayers of cells in 6 well plates were co-cultured with 10 adult liver flukes that had been subjected to RNA interference to silence expression of *Ov-grn-1* (below) in the upper chamber of Transwell (4 μm pore size) inserts (Corning, USA).

### Fluorescence microscopy

Recombinant r*Ov-*GRN-1 and rTRX (control) proteins were amine labeled with Alexa Fluor 488 (AF488—Life Technologies) [57]. H69 cholangiocytes were grown to 50% confluence on optical quality glass bottomed culture dishes containing a 0.17 mm thick cover glass (World Precision Instruments). AF488-labeled proteins were added to cells at a final concentration of 3 μM and incubated for 18 h at 37°C under 5% CO_2_. Cells were fixed in 4% paraformaldehyde/PBS for 20 min at room temperature. Cells were permeabilized in 0.1% Triton X-100/PBS and stained with 10 μM DAPI and 165 nm Alexa Fluor 568 Phalloidin (Life Technologies). Specimens were mounted in 5% N-propyl-Gallate (Sigma) in 80% glycerol/PBS. For localization studies, cells were fixed in 4% paraformaldehyde in PBS for 20 min at room temperature and then permeabilized in 0.1% Triton X-100/PBS. Fixed and permeabilized cells were probed with either LAMP1 (lysosomes), Rab5, EEA1 (early endosome), Rab7 (late endosomes), GRP78 BiP (endoplasmic reticulum) or anti-golgin97 (Golgi) antibodies at a 1:200 dilution, followed by incubation with Alexa Fluor 568 goat anti-mouse or Alexa Fluor 568 goat anti-rabbit antibodies at a 1:1000 dilution. Conventional fluorescence imaging was performed with a 60× (NA1.4) objective using an A1 confocal research microscope (Nikon) or a DeltaVision personal research microscope (Applied Precision, GE Healthcare). Super Resolution imaging was performed using a DeltaVision OMX 3D-Structured Illumination Imaging system (Applied Precision, GE Healthcare) as previously described [58] and images were processed as described elsewhere [59].

### Chorioallantoic membrane assay

The chorioallantoic membrane assay (CAM) assay was established based on previous studies using quail eggs [21,60]. Briefly, fertilized eggs of the quail *Cortunix cortunix* were incubated at 37°C in a humidified incubator for five days. The surface of the eggshell was sanitized by wiping with 70% ethanol. Subsequently, a 0.5-cm square window of shell was surgically resected. The CAM with visible blood vessels was gently pulled down after which the window was sealed with clear tape. Eggs were incubated at 37°C for 18 h. Subsequently, filter paper presoaked in 20 μl of 2 or 20 pMoles of r*Ov*-GRN-1 was implanted. The surgical window was resealed, and the eggs incubated at 37°C for 18 h. Eggs were chilled and the surgical window was fixed with 25% glutaraldehyde. Implanted filter papers were trimmed and washed with PBS prior to counting the blood vessels using an Olympus SZX12 dissecting microscope with a light box using 32× magnification.

### Mouse wounding assay

A head biopsy model was employed, as recommended for assessment of growth factors in wound healing [18,19]. Briefly, five female BALB/c mice per group (rTRX, PBS and r*Ov-*GRN-1) were anesthetized (intraperitoneal xylazine 16 mg kg^-1^; ketamine 80 mg kg^-1^), after which the crown of the head was shaved with an electric razor. Mice were anesthetized three days later and the surgical site was sterilized with 70% ethanol wipes. A skin-deep wound of 5 mm in diameter was inflicted on the crown of the head using biopsy punch (Zivic instruments). The lesion was rinsed with antiseptic (Betadine, Sanofi), after which 56 pMoles of r*Ov*-GRN-1, rTRX or PBS suspended in 1.5% methyl cellulose (Sigma) in 50 μl was applied. Thereafter, the lesion was covered with Elastoplast Spray Plaster (Beiersdorf). Progress of the wound, and wound closure, was documented with photographs taken at cumulative 1.6× magnification **using** a dissection microscope (Olympus) fitted with a Nikon D200 camera, each day for five days. Wound closure was ascertained in an unblinded fashion by comparison of the surface area of the lesion with the size as documented immediately after the wound was inflicted, with the assistance of ImageJ software.

### Relative/absolute quantitation labeling of cellular proteins using isobaric tags

H69 cholangiocytes were cultured in complete medium until ~50% confluence was reached in T75cm^2^ flasks. Cells were washed three times in PBS, 13.5 ml of low nutrient medium was added and cells were grown overnight at 37°C in 5% CO_2_. r*Ov*-GRN-1 or rTRX (500 nM) were prepared in pre-warmed low nutrient media and 1.5 ml was added to each flask for a final concentration of 50 nM recombinant protein in media. Cells were grown for 0.5, 1, 4, 8, 16, 24 and 48 h, washed 3× in PBS and snap frozen then stored at -80°C. Cells were lysed in three ml of 0.2% SDS with 3× freeze/thaw cycles and centrifuged at 4000 *g* to remove cell debris. The protein in the supernatant was precipitated with methanol [61]. Precipitated protein was prepared as per manufacturer’s instructions from the 8-plex iTRAQ [62] kit (AB SCIEX) as previously described [63]. Briefly, 100 μg of protein samples for each time-point were digested with 2 μg of trypsin (Sigma-Aldrich) at 37°C for 16 h. Each sample was labeled with different iTRAQ labels and was subsequently combined into one tube for OFFGEL fractionation and LC-MS/MS analysis.

### Peptide OFFGEL fractionation, mass spectrometry and protein identification

A 3100 OFFGEL Fractionator (Agilent Technologies) with a 24 well setup was used for peptide separation based on isoelectric point (pI), as described [64]. Sample clean up and desalting were performed using a HiTrap SP HP column (GE Healthcare) and a Sep-Pak C18 cartridge (Waters). Samples were separated with the OFFGEL Fractionator and collected fractions were desalted using ZipTip (Millipore) followed by evaporation by centrifugation under vacuum. The sample was reconstituted, desalted and separated with an analytical nano-HPLC column (150 mm x 75 μm 300SBC18, 3.5 μm, Agilent Technologies) before being applied to a Triple TOF 5600 mass spectrometer (Applied Biosystems); the results were analyzed as described [64].

### Bioinformatic analysis of proteomic sequence data

Database searches were performed on the SwissProt database (version September 2013) using MASCOT search engine v4.0 (Matrix- Science) with parameters as previously described [64]. Findings from Mascot searches were validated using the program Scaffold v.4.2.1 (Proteome Software Inc.) [65]. Peptides and proteins were identified using the Peptide Prophet algorithm [66], using a probability cut-off of 95% (peptides) or 99% probability (proteins), and contained at least two identified peptides (proteins) [67]. Proteins containing similar peptides that could not be differentiated based on tandem mass spectrometry (MS/MS) analysis were grouped to satisfy the principles of parsimony. A false discovery rate (FDR) of <0.1% was calculated using protein identifications validated using Scaffold v.4.2.1. Furthermore, a FDR of <0.4% for the proteins identified was calculated using protein identifications validated by Scaffold. Proteins sharing significant peptide evidence were grouped into clusters. Channels were corrected in all samples according to the algorithm described in i-Tracker [68]. Acquired intensities in the experiment were globally normalized across all acquisition runs. Individual quantitative samples were normalized within each acquisition run, and intensities for each peptide identification normalized within the assigned protein. The reference channels were normalized to produce a 1:1 fold change. Normalization calculations were performed using medians to multiplicatively normalize data. A protein-protein interaction analysis was performed using the String software (http://string-db.org/) based on compiled available experimental evidence [69].

### RNA interference

Adult flukes from hamsters were transformed with *Ov-grn-1* targeted dsRNA (residues 49–333 of the 444 nucleotide transcript [7]) by square wave electroporation [70]. Briefly, 20 flukes in 100 μl of RPMI 1640 medium were dispensed into a 4 mm gap electroporation cuvette containing 5 μg dsRNA followed by a square wave pulse of 125 volts of 20 milliseconds duration. Transformed parasites were cultured for 1, 2, 3, 5 and 7 days after treatment. Total RNA was isolated from parasites and *Ov-grn-1* expression measures using qRT-PCR with SYBR Green (TAKARA Perfect Real-time kit, Japan) and *O*. *viverrini* actin (GenBank EL620294.1) as a reference transcript [70]. The mRNA levels of *Ov-grn-1* were normalized to actin mRNA and are presented as the unit value of 2-ΔΔCt where ΔΔCt = ΔCt (treated worms)– ΔCt (control, luciferase dsRNA-treated worms) [70,71]. ES products from treated and control worms were collected and tested for cell proliferation activity (above). The time point at which maximum cell proliferation was attained with ES products from *Ov-grn-1* ds-RNA-treated flukes was used to calculate the percent reduction in cell proliferation relative to ES products from *luc* dsRNA-treated flukes. ES products from dsRNA-treated (*Ov-grn-1* and *luc*) worms were assessed by SDS-PAGE with silver staining to ensure that the protein profiles were consistent between treatments.

### Gene arrays

Specific gene pathways in H69 cholangiocytes exposed to r*Ov*-GRN-1 as described above were investigated by qRT-PCR. Cells from 6-well plates were harvested employing a cell scraper after 1 h (“early” time point) or 24 h (“late” time point) after the addition of recombinant proteins, and total RNA was isolated using the miRNeasy Mini Kit (Qiagen). The concentration, purity and integrity of the RNA were evaluated using spectrophotometry (Nanodrop 1000) and an Agilent 2100 Bioanalyzer. The RNAs were stored at -80°C until processed for cDNA synthesis and qPCR following the RT^2^ Profiler PCR Array protocol (Qiagen). Four RT^2^ Profiler PCR Arrays (Qiagen) were screened—Wound healing (PAHS-121Z); Oncogenes and Tumor Suppressor genes (PAHS-502Z); Epithelial-Mesenchymal Transition (EMT) (PAHS-090Z); Toll-like Receptors (TLR) (PAHS-018Z). Ct values were exported and analyzed for significance using RT^2^ Profiler PCR Array Data Analysis software version 3.5 (http://pcrdataanalysis.sabiosciences.com/pcr/arrayanalysis.php). The relative quantitation, included in the software, was performed using the 2^-ΔΔCt^ method employing a panel of 5 house keeping genes as follows: beta actin (NM_001101), beta-2-microglobulin (NM_004048), glyceraldehyde-3-phosphate dehydrogenase (NM_002046), hypoxanthine phosphoribosyltransferase 1 (NM_000194), and ribosomal protein, large, P0 (NM_001002). Control groups (cells exposed to media alone) were used as calibrator samples. Three biological replicates were assessed and included in the analysis. The qPCR experiments were performed using a Bio-Rad iCycler iQ5 with an initial activation step of 95°C for 10 min followed by 40 cycles of 95°C for 10 sec and 60°C for 1 min. A melting curve analysis from 55°C to 95°C and 0.5°C temperature increment every 30 sec was included at the end of the run.

### Statistical analyses

Statistical analyses were conducted using GraphPad Prism 6.02 software. For cell proliferation studies, two-way ANOVA with Sidak’s multiple comparison tests were used to compare the changes in proliferation induction of ES products from *Ov-grn-1-* compared to *luc-*dsRNA treated flukes. Degrees of freedom for the *F*-test output of the ANOVA were calculated with DFn and DFd representing the degrees of freedom of the numerator and denominator, respectively. For CAM studies, statistical analysis compared treatment (r*Ov*-GRN-1) and media alone controls using one-way ANOVA with Dunnett’s correction for multiple comparisons. For wound healing studies, closure rate of wounds was compared by 2-way ANOVA with Dunnett’s correction for multiple comparisons. For proteomics studies with cell lines, differentially expressed proteins were determined using Kruskal-Wallis Test and results were expressed in log2 ratios. Proteins with a *P*-value < 0.05 and a significant log2 fold-change >0.6 or <-0.6 (for upregulated and downregulated proteins respectively) were considered in subsequent analyses. For gene expression studies, the fold change values of the genes from the four analyzed gene arrays were exported to GraphPad Prism 6.02, pooled and plotted in a volcano plot and the significantly dysregulated genes (*P* ≤ 0.05) plotted as a gene expression heatmap using Microsoft Excel.

## Supporting Information

S1 Fig r*Ov*-GRN-1 but not rTRX is internalized by cholangiocytes; related to Fig 1. **(A)** Relative fluorescence units from a titration of Alexa Fluor 488 (AF)-labeled recombinant r*Ov-*GRN-1-AF and thioredoxin (rTRX-AF). **(B)** Fluorescence images of human H69 cholangiocytes with nuclear stain (blue) after 16 hours of co-culture with 3 μM r*Ov*-GRN-1-AF (green). **(C)** As for panel (B) but with 3 μM rTRX-AF (green). **(D)** Negative control without AF-labeled protein. **(E)** Quantification of fluorescence per cell from panels B-D performed with AX10 software. ****P<0.0001.
(TIF)

S2 Fig Recombinant *Ov*-GRN-1 internalized by cholangiocytes does not localize to major cellular organelles. Fluorescence images and 2D histograms showing the corresponding pixel intensities within the cell volume for the red and green channels of human H69 cholangiocytes after 16 hours of co-culture with 3 μM r*Ov*-GRN-1-AF (green), DAPI nuclear stain (blue) and the organelle–specific markers (red) LAMP-1 for lysosomes (A, B), anti-EEA1 for early endosomes (C, D), anti-Golgin97 for golgi (E, F), and anti-GRP78 for endoplasmic reticulum (G, H).
(TIF)

S3 Fig Silencing of *Ov-grn-1* gene expression results in significantly reduced capacity of fluke ES products to drive proliferation of cholangiocytes; related to Fig 2. **(A)** qPCR validation of *Ov-grn-1* knockdown. SYBR green real time PCR used to quantify *Ov-grn-1* transcript levels relative to controls electroporated with double stranded luciferase (*luc*). Day 0 is untreated control. **(B)** ES products (10 μg/ml) from *Ov-grn-1* dsRNA-treated flukes have a significantly reduced capacity to drive proliferation of H69 cholangiocytes compared to *luc* dsRNA control worms. Mean values ± SEM of three biological replicates; **, *P* < 0.01.
(TIF)

S4 Fig Comparison of Excretory/Secretory S products from adult *O*. *viverrini* treated with double stranded RNAs for *Ov-grn-1* or luciferase (*luc*) after 1, 3 and 5 days of *in vitro* culture. SDS-PAGE gels were stained with silver. Protein profiles were consistent between samples on each day of sampling.
(TIF)

S1 Table Validation of cholangiocyte proteins for which expression levels changed significantly after exposure to r*Ov*-GRN-1; related to Fig 4. (XLSX)

S2 Table Cholangiocyte proteins that underwent significantly regulated expression by >50% after exposure to *Ov*-GRN-1; related to Fig 4. This data forms the basis of Fig 4. Values in log2, ±0.6 = fold change ±50%.
(XLSX)

S3 Table Changes in cholangiocyte gene expression in response to exposure to r*Ov*-GRN-1; related to Fig 4. (XLSX)

S4 Table Genes for which expression levels changed significantly after exposure to *Ov*-GRN-1; related to Fig 4. (XLSX)

S1 Movie Fly-through video depicting *Ov*-GRN-1 internalized within a cultured human H69 cholangiocyte; related to Fig 1. Rendered from 3D-SIM lateral (xy) and axial (yz) overview images of a well-separated individual cholangiocyte viewed in Fig 1C and 1D showing r*Ov*-GRN-1 (green) present between the apical and basal actin filaments (red) of the cholangiocyte. The nucleus is stained blue.
(MOV)
