# Supplementary figures and images for: Carcinogenic Parasite Secretes Growth Factor That Accelerates Wound Healing and Potentially Promotes Neoplasia
Source: PLoS Pathog. 2015 Oct 20;11(10):e1005209. doi: 10.1371/journal.ppat.1005209 (PMC4618121; doi:10.1371/journal.ppat.1005209)

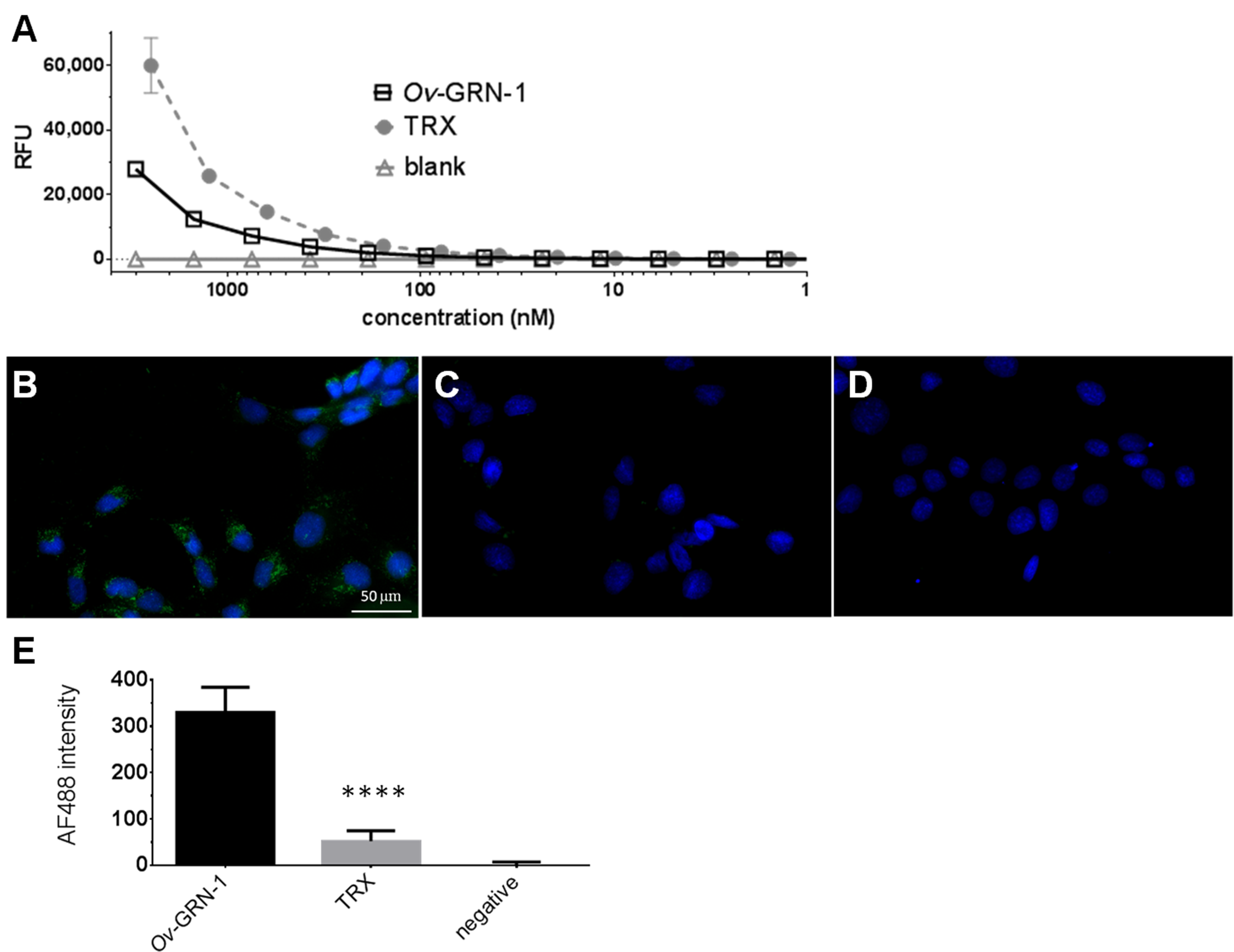

Supplement: S1 Fig — (A) Relative fluorescence units from a titration of Alexa Fluor 488 (AF)-labeled recombinant rOv-GRN-1-AF and thioredoxin (rTRX-AF). (B) Fluorescence images of human H69 cholangiocytes with nuclear stain (blue) after 16 hours of co-culture with 3 μM rOv-GRN-1-AF (green). (C) As for panel (B) but with 3 μM rTRX-AF (green). (D) Negative control without AF-labeled protein. (E) Quantification of fluorescence per cell from panels B-D performed with AX10 software. ****P<0.0001. (TIF) [file ppat.1005209.s001.tif]

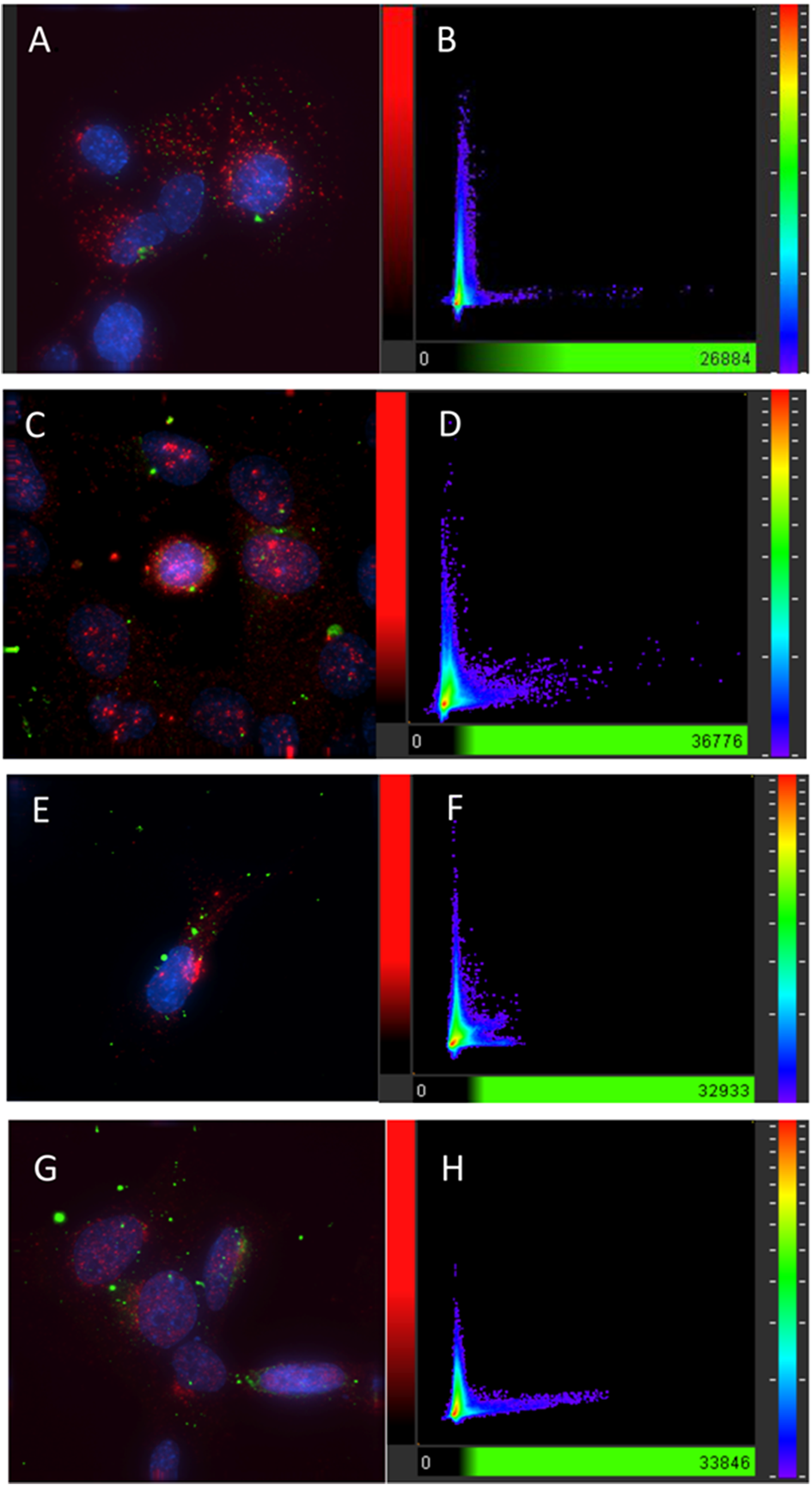

Supplement: S2 Fig — Fluorescence images and 2D histograms showing the corresponding pixel intensities within the cell volume for the red and green channels of human H69 cholangiocytes after 16 hours of co-culture with 3 μM rOv-GRN-1-AF (green), DAPI nuclear stain (blue) and the organelle–specific markers (red) LAMP-1 for lysosomes (A, B), anti-EEA1 for early endosomes (C, D), anti-Golgin97 for golgi (E, F), and anti-GRP78 for endoplasmic reticulum (G, H). (TIF) [file ppat.1005209.s002.tif]

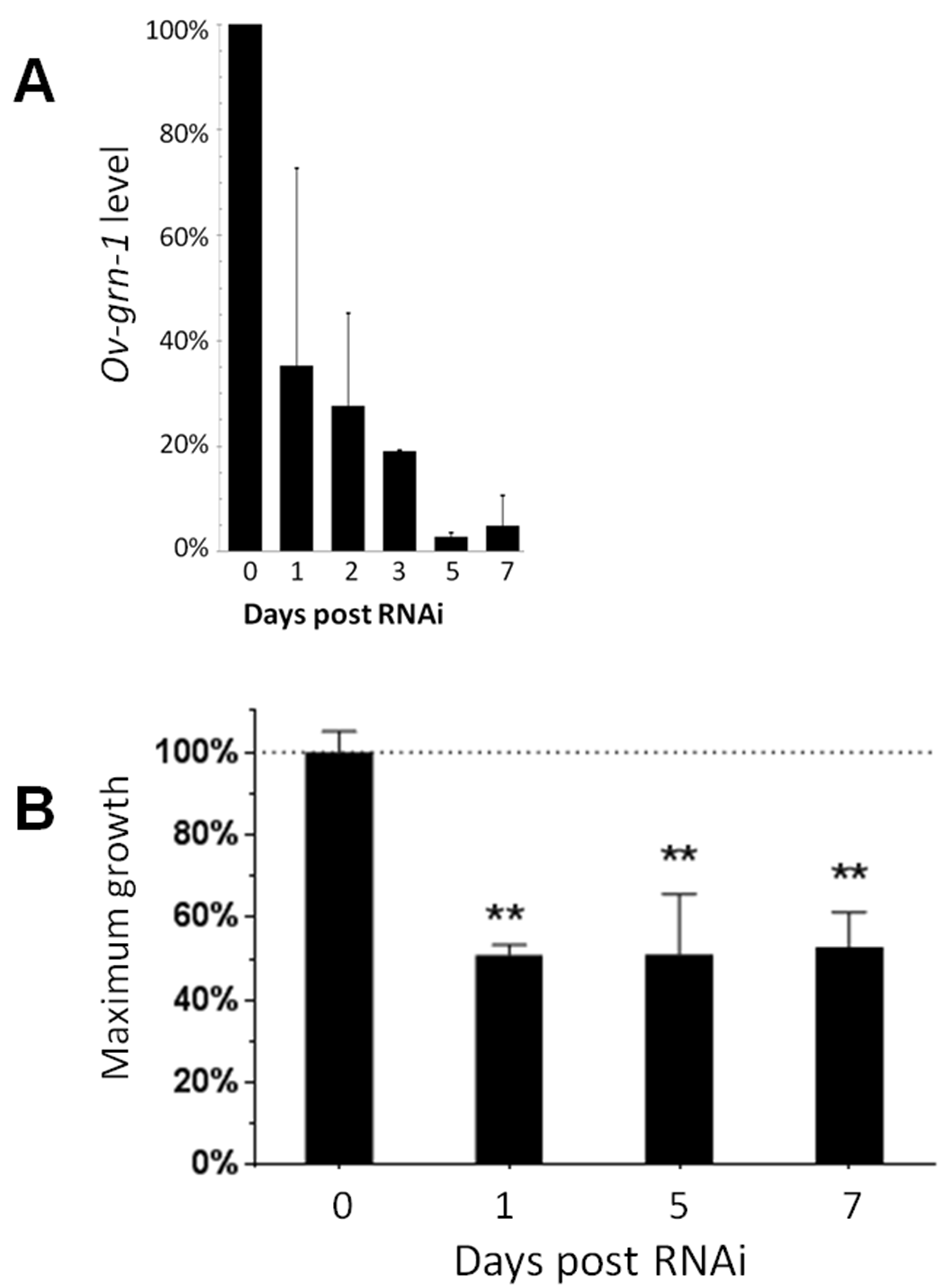

Supplement: S3 Fig — (A) qPCR validation of Ov-grn-1 knockdown. SYBR green real time PCR used to quantify Ov-grn-1 transcript levels relative to controls electroporated with double stranded luciferase (luc). Day 0 is untreated control. (B) ES products (10 μg/ml) from Ov-grn-1 dsRNA-treated flukes have a significantly reduced capacity to drive proliferation of H69 cholangiocytes compared to luc dsRNA control worms. Mean values ± SEM of three biological replicates; **, P < 0.01. (TIF) [file ppat.1005209.s003.tif]

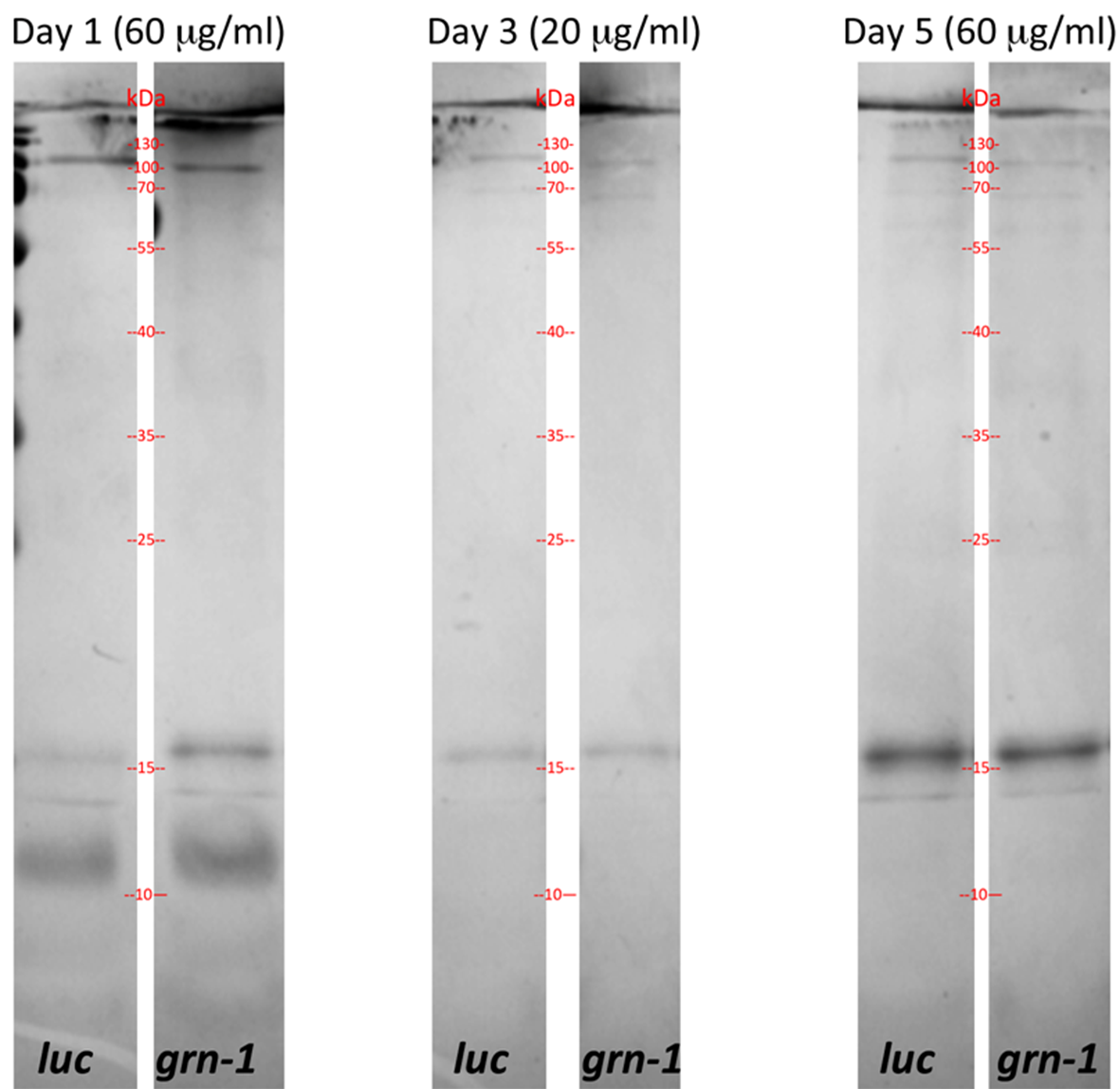

Supplement: S4 Fig — SDS-PAGE gels were stained with silver. Protein profiles were consistent between samples on each day of sampling. (TIF) [file ppat.1005209.s004.tif]
